# Cryopreserved amniotic membrane in chronic nonhealing wounds: a series of case reports

**DOI:** 10.1007/s10561-023-10100-5

**Published:** 2023-11-09

**Authors:** Vojtech Horvath, Alzbeta Svobodova, Joao Victor Cabral, Petr Stadler, Jaroslav Lindner, Miluse Berka Mrstinova, Lukas Balogh, Katerina Jirsova

**Affiliations:** 1 Department of Vascular Surgery, Na Homolce Hospital, Prague, Czech Republic; 2 2nd Department of Surgery-Department of Cardiovascular Surgery, First Faculty of Medicine, Charles University and General University Hospital in Prague, Prague, Czech Republic; 3 Department of Obstetrics and Gynaecology, 2nd Faculty of Medicine, Charles University and Motol University Hospital, Prague, Czech Republic; 4 Laboratory of Biology and Pathology of the Eye, Institute of Biology and Medical Genetics, 1st Faculty of Medicine and General Teaching Hospital, Charles University, Albertov 4, 128 01 Prague, Czech Republic; 5 Department of Transplantation and Tissue Bank, Motol University Hospital, Prague, Czech Republic

**Keywords:** Cryopreserved amniotic membrane, Wound healing, Chronic nonhealing wound, Chronic venous insufficiency, Peripheral artery disease

## Abstract

A case series of the use of amniotic membrane (AM) for treating chronic nonhealing wounds. It presents five cases of polymorbid patients with a total of nine chronic nonhealing wounds. The patient group consisted of four men and one woman with various comorbidities, aged 45–72 years. The mean initial wound size was 15.8 cm^2^, and the mean time from the onset of the wound to the first application of AM was 122 weeks. The wounds were caused by chronic venous insufficiency and/or peripheral arterial disease. Wounds were treated in a standardized protocol. AM was applied weekly in the first month and then every two weeks. Photo documentation of the wound and microbiological colonization was carried out at each visit. In three out of five patients, the AM treatment effectively promoted healing up to complete wound closure. In two cases, the wounds stayed unhealed despite numerous AM applications. Pain relief was noted in all patients. The success of the treatment was closely tied to patient factors, such as adherence to the prescribed treatment regimen and individual patient characteristics. In some cases, treatment failure was observed, possibly due to underlying comorbidities, wound parameters, or poor patient compliance. AM treatment has the potential to become a viable treatment option for these nonhealing wounds. However, the effectiveness of the treatment may be influenced by various patient factors and the underlying cause of the wound. Therefore, it is crucial to have an individualized treatment plan that considers these particular factors.

## Introduction

Chronic nonhealing wounds (NHW) are a growing concern across the globe, with an increasing number of patients affected each year (Jarbrink et al. 2016). These wounds are most commonly found in the lower leg, specifically below the knee, and can be caused by a variety of factors, such as chronic venous insufficiency (CVI), peripheral artery disease (PAD), and diabetes mellitus (DM). Other contributing factors include complicated surgical wounds, injuries, and pressure ulcers (Ahmajarvi et al. 2019).

The treatment of chronic wounds is concentrated in the ambulatory sector, where it accounts for almost 15% of primary diagnosis costs and contributes to another 40% of outpatient care costs as an associated diagnosis (Nussbaum et al. 2018). Due to the need for long-term care, patients of all ages can experience reduced quality of life, resulting in substantial costs to the healthcare system and society in terms of lost productivity and reduced well-being (Krupová and Pokorná 2020).

Effective wound healing is the ultimate goal after stabilizing the primary cause. However, many patients do not achieve satisfactory improvement even after several months of intensive ambulatory care using “best medical practice” methods in specialized centers. This may be due to cellular and molecular repair mechanism changes in chronic wounds (Martin and Nunan 2015). Since standard treatment of NHW requires significant personnel and material resources, alternative treatments are being recently utilized (Al-Gharibi et al. 2018). The burdens placed on patients and the healthcare system may be lessened through cost reduction and improved cost-effectiveness (Tapking et al. 2020). Determining systemic trends of care that may be used to model and enhance care, for instance, may be another aim that goes beyond any specific patient, such as reducing readmission rates due to wound recurrence and infection.

One of the most promising options in NHW treatment is the amniotic membrane (AM), which contains several bioactive molecules that are responsible for its anti-inflammatory (Navas et al. 2018), anti-fibrotic (Mao et al. 2021), antibacterial (King et al. 2007) and analgesic properties (Svobodova et al. 2023a). AM has emerged as a possible treatment option for ophthalmology, oral surgery, and chronic nonhealing wounds (Jirsova and Jones 2017; Odet et al. 2021). Currently, cryopreserved and dehydrated (freeze-dried) forms of AM are available for clinical use. Studies have shown that cryopreserved AM stimulates epithelial cell migration and positively regulates vascularization (Zelen et al. 2015; Serena et al. 2015).

The use of AM in wound healing is an exciting area of research with the potential to revolutionize the treatment of chronic wounds, particularly in cases where conventional treatment options have failed. Our team previously published a study demonstrating cryopreserved AM treatment’s positive effects (Svobodova et al. 2022). As research continues, we hope to see more effective and innovative treatment options emerge for patients suffering from chronic nonhealing wounds.

## Material and methods

The study followed the Ethics Committee’s standards of all participating institutions (1st Medical Faculty of Charles University, General Teaching Hospital, University Hospital Motol, and Na Homolce Hospital, all in Prague) and adhered to the tenets set out in the Declaration of Helsinki.

### Amniotic membrane allografts preparation

Cryopreserved AM allgrafts were prepared in Tissue Bank, Motol University Hospital, Prague, as described previously (Svobodova et al. 2022). Shortly graft containing an intact amniotic membrane was aseptically processed from placentas donated by healthy screened mothers (medical record and personal history was evaluated to prevent the transmission of infectious diseases) having a caesarian section. AM was decontaminated by antibiotic solution (Base 128, Alchimia), then minimally processed (cleaned from blood clots) and cryopreserved (− 80 °C) in a mixture of DMEM and glycerol 1:1 on Sanatyl tissue (Tylex, Letovice, Czech Republic) support. During tissue processing and packaging, sterility tests were performed. Only allografts with negative serology (HIV, hepatitis B and C, syphilis) both at the day of tissue retrieval and 180 days after were released for grafting.

Here we present a set of five case reports from our registry, upon which we intend to demonstrate the efficacy of AM. All patients included in this study fulfill the required inclusion criteria: age over 18 years, presence of nonhealing wound lasting longer than 6 weeks, without any plans for surgery/intervention for underlying disease, and signed informed consent. Our patient group consisted of four men and one woman with numerous comorbidities, aged 45–72 years, and with a body mass index (BMI) of 27.2–36.8. The mean initial wound size was 15.8 cm^2^ (1.2–54.5 cm^2^), and the mean time from the onset of the wound to the first application of AM was 122 weeks (from 9 to 267 weeks). The etiology of the wounds was CVI (three cases) and PAD (two cases). The primary pathology leading to wound formation was treated before the monitored period. Emphasis was placed on the proven chronicity of the wounds, with an average time from documented wound onset to the first AM application of 121.8 weeks (median 41.5 weeks, from 9 to 267 weeks).

### Patients and treatment

All patients were treated according to the protocol that we described previously (Svobodova et al. 2022). Briefly, blood samples were taken (complete blood count, urea, creatinine, C-reactive protein, albumin, total protein, and glycosylated hemoglobin for patients with diabetes mellitus). Patients with diabetes underwent transcutaneous oximetry near the wound, while patients without diabetes underwent ankle-brachial index measurement as a non-invasive evaluation of limb circulation. A separate bacterial swab was collected from all wounds for bacteriological testing. In cases of massive bacterial growth, targeted antibiotic therapy was initiated.

All patients were primarily treated in a specialized center for at least six weeks using modern wound dressing methods. The primary coverage was chosen according to the condition of the wound and the amount of exudate. The combination most often used was gel covering containing sodium alginate/hypochlorite and sodium hypochlorite (NU-GEL™/Granudacyn gel) together with a layer of absorbent covering based on hydrofiber containing silver ions (Exufiber Ag + or AQUACEL® Ag). Shallow defects were covered with a foam cover with a silver-containing silicone layer (Mepilex® Ag) to maintain negative cultures or reduce the bacterial load. The wound treatment protocol using AM was followed during each dressing change, with wound debridement, measurement, and photography recorded. Simultaneously subjective pain degree was evaluated using a visual analog score on a scale from 0 (no pain) to 10 (the worst pain).

The AM, thawed to room temperature and rinsed with saline solution, was applied to the entire wound defect in a single layer once a week. After the first four weeks and based on the wound’s response, subsequent applications were scheduled every two weeks. A foam dressing with a contact silicone layer (Mepilex® XT) was used as the fixative secondary dressing during the application period. During the inter-application period, the primary dressing was chosen individually based on the current wound state. A bandage covering the entire limb was applied during each dressing change in patients with CVI etiology. The AM was left on the wound for at least 48 h, and the secondary dressing was changed as needed according to exudate strength.

## Results

### Case report 1

A 60-year-old male with two ulcers on the left lower extremity caused by chronic venous insufficiency (CVI). The defects (D1 and D2) were treated in a local dermatology ambulance for 37 weeks, and the patient was not evaluated for potential vascular defects.

The condition was complicated by a bullous erysipelas infection on the left lower extremity, leading to a septic shock and a diagnosis of aortic valve infective endocarditis. As a result, the patient was urgently indicated for an aortic valve replacement, cryoablation, and left atrial appendage closure. After that, in one hospitalization for the treatment of CVI (ligation of perforator veins) to heal the defect and eliminate the infectious focus.

**Fig. 1:**
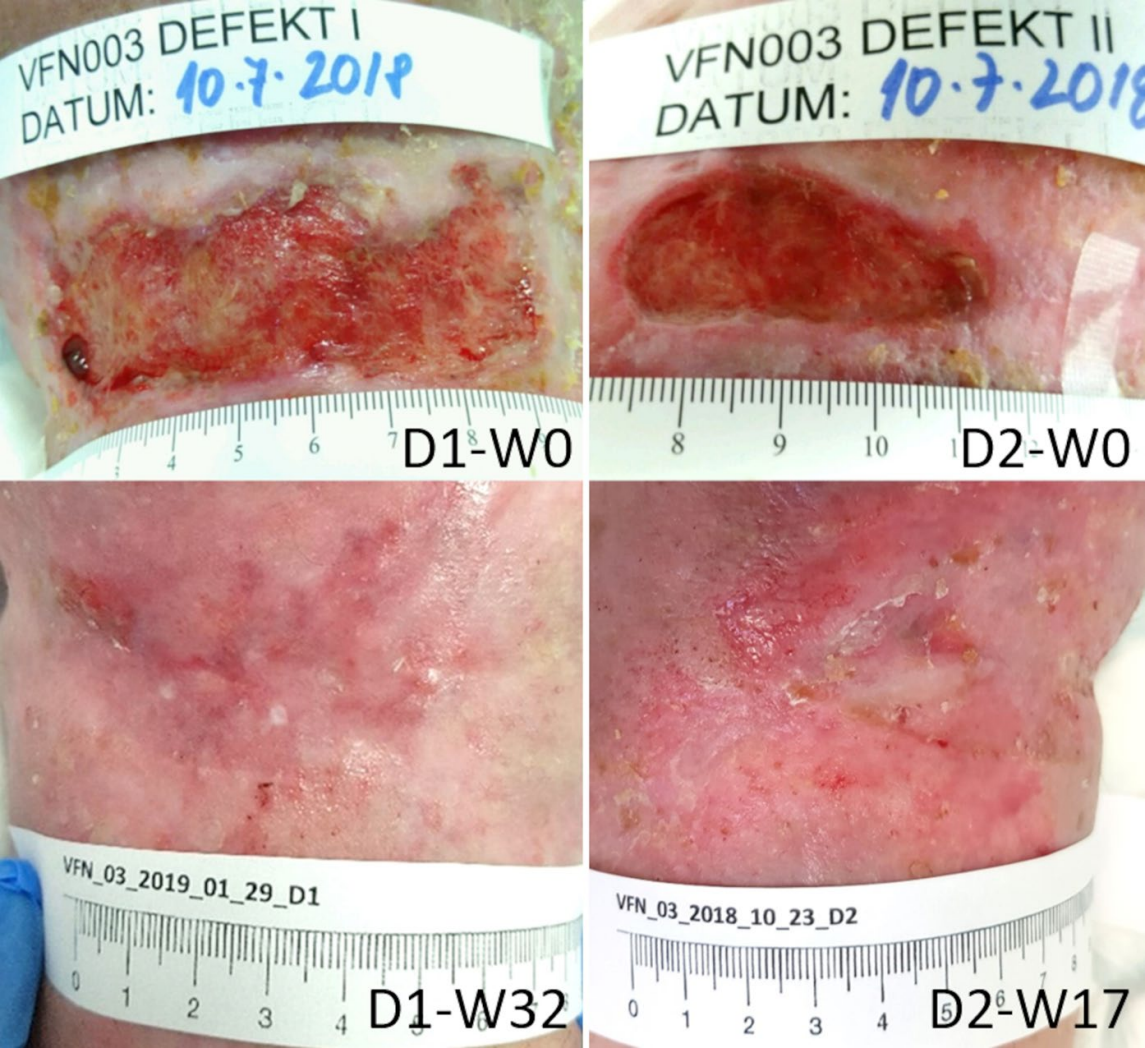
CASE 1. Defects D1 (medial ulcer) and D2 (lateral ulcer) before and after amniotic membrane applications for 32 and 17 weeks (W), respectively

After discharge, the patient was treated with moist wound healing (Aquacel®Ag) and appropriate compression therapy in the outpatient care in the dedicated center, which resulted only in a minimal tendency for healing. When included in the AM study group, the ulcers were 16.8 cm^2^ and 4.5 cm^2^ in size, Fig. 1, colonized by beta-lactamase-negative *Staphylococcus aureus* and *Morganella morganii*. Ankle pressures did not indicate limb-threatening ischemia, inflammatory markers were low, and the nutritional status was satisfactory.

**Fig. 2:**
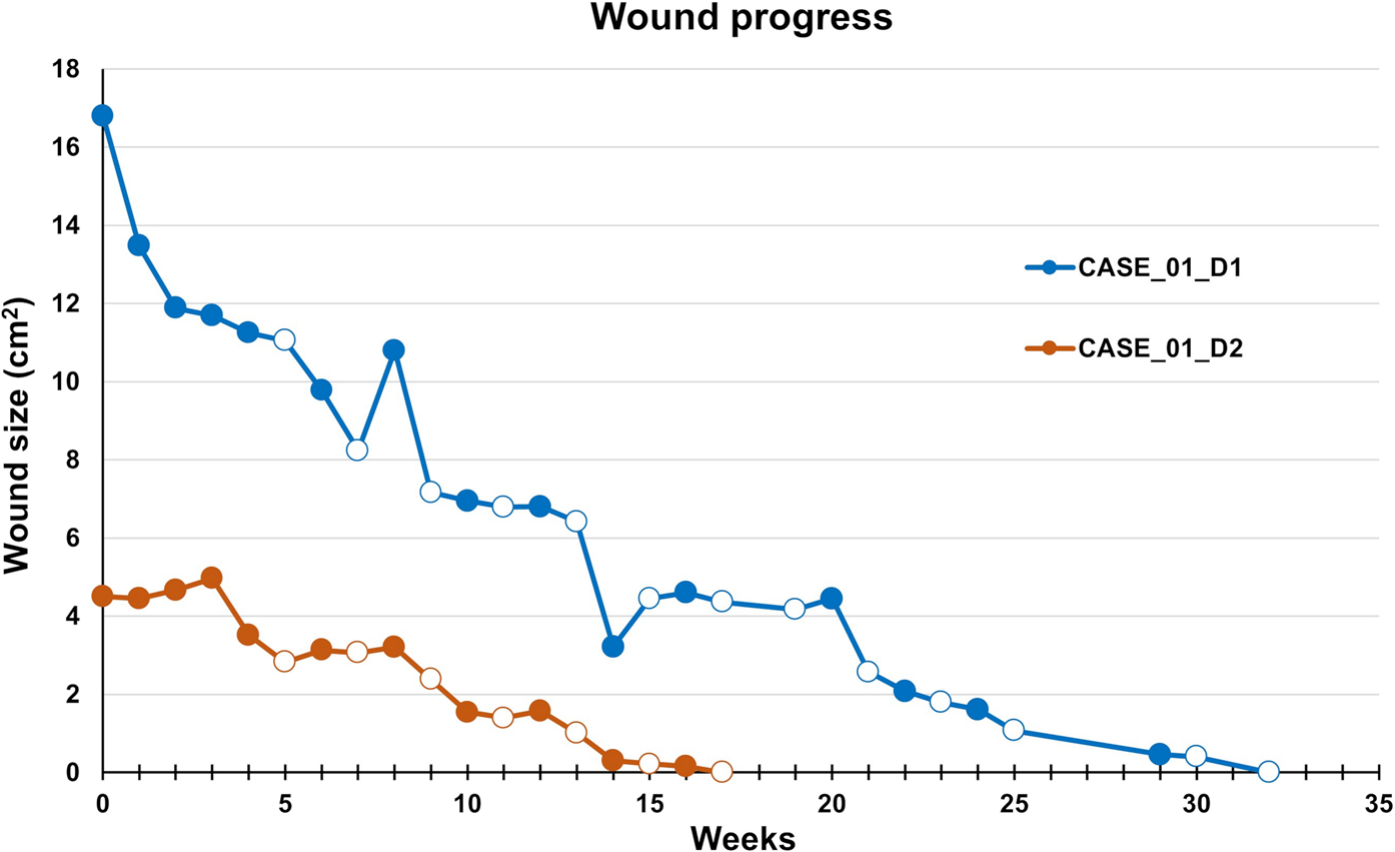
CASE 1. The progress of the wound size closure of the patient 1. Closed and open markers reflect visits with or without amniotic membrane application, respectively

The wounds were managed according to the specified protocol, regularly monitoring the wound’s microbiological colonization. Defect D1 was completely healed after 32 weeks (with 15 applications of AM), and defect D2 after 17 weeks (with 11 applications of AM), with a very satisfactory wound area reduction progress, Fig. 2. The pain decreased by half after the first week of AM application and completely disappeared after the 10th week of treatment. No local adverse effects were observed after AM application. The wounds remained healed during the six months follow-up period.

### Case report 2

A 72-year-old male was admitted for AM application for a semicircular ulcer on the left calf (D1), resulting from insufficiency of the great saphenous vein (GSV) and calf perforators. The patient was initially treated in a different center and was not assessed vascularly. In the personal history, the patient presented chronic renal insufficiency, arterial hypertension, chronic atrial fibrillation requiring anticoagulation, and compensated type II diabetes mellitus.

**Fig. 3:**
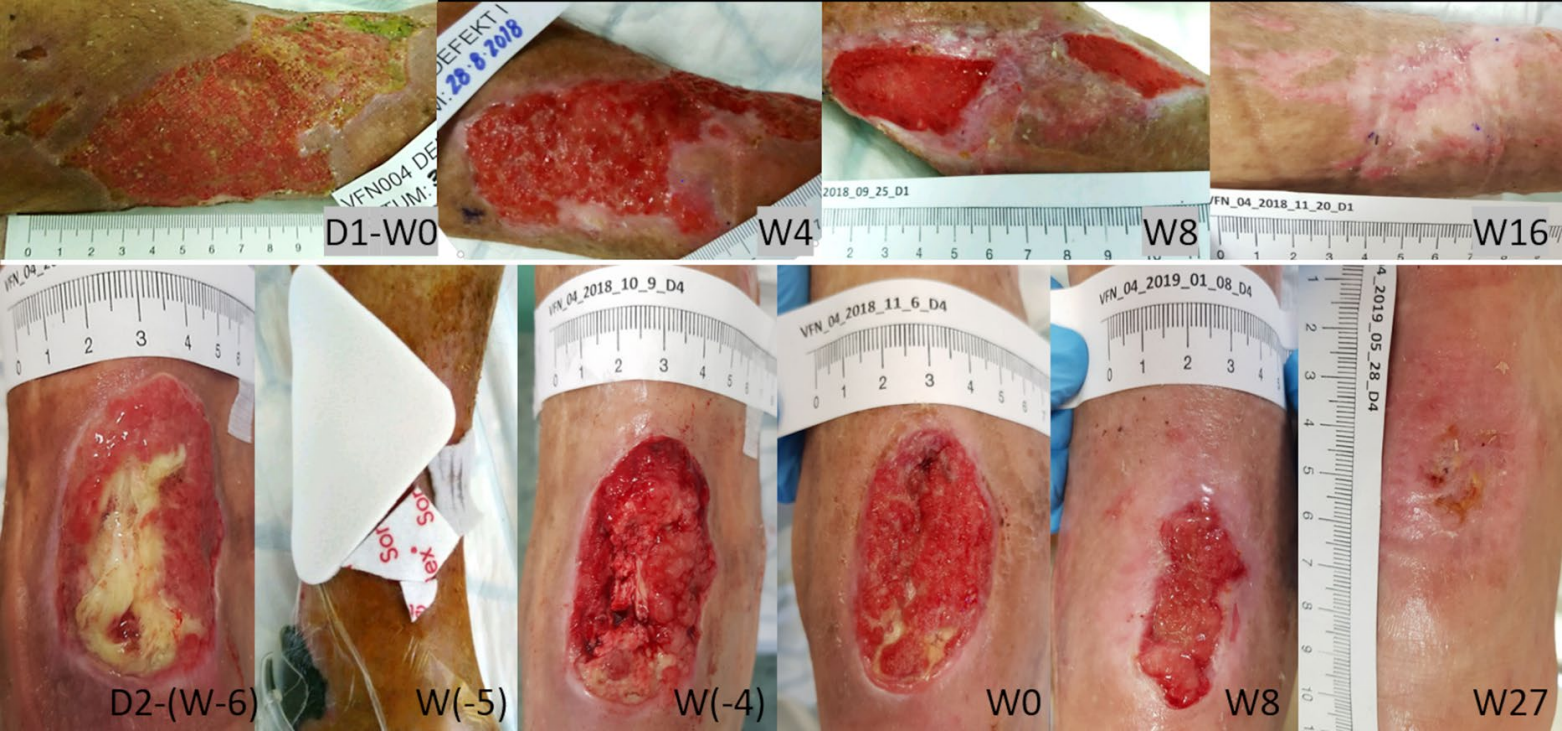
CASE 2. Semicircular ulcer (defect D1) of the left leg before (week 0, W0) the first amniotic membrane application and throughout the therapy (W4–W16). Defect of the Achilles tendon (D2), the treatment before (W-6 to W0) the first amniotic membrane application, and during the therapy (W0–W27)

After endovascular treatment of the GSV and perforators, only minimal reduction in the defect size was observed during 6 weeks of treatment, despite regular outpatient dressings (Aquacel®Ag, Nu-Gel™) with appropriate compression therapy. At enrollment, the ulcer surface area was 50.5 cm^2^, and the wound had persisted for nine weeks (Fig. 3, defect D1); besides, it was colonized by *Proteus mirabilis*, *Escherichia Coli*, and *Streptococcus dysgalactinae*. Given the history of diabetes, transcutaneous oximetry was performed during the initial examination, and glycated hemoglobin (HbA1c) was added to the standard panel of laboratory tests. All results were satisfactory.

**Fig. 4:**
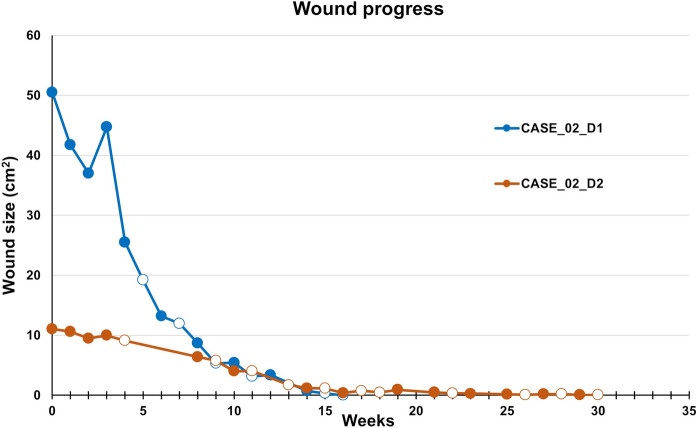
CASE 2. The healing progress of the wound size closure of the patient 2. Closed and open markers reflect visits with or without amniotic membrane application, respectively

The patient was treated according to the protocol. The defect was completely healed after 14 weeks (10 applications of AM). The application was performed without any local adverse effects. After the first week of AM application, the patient reported significant pain relief (decrease from grade 5 to 3), and from the fifth week of therapy, the patient no longer perceived the pain. During the follow-up period, the patient developed a pressure ulcer, Fig. 3, D2 defect in the area of the Achilles tendon due to unsuitable footwear, the tendon was exposed, and the defect was colonized by *Methicillin-resistant Staphylococcus aureus* (MRSA). This condition required hospitalization with partial tendon resection and negative pressure wound therapy application. After stabilization of the defect and eradicating MRSA, the patient was equipped with a vacuum fixation plate (VACO®ped) to ensure the tendon’s resting mode while allowing regular bandages. After that, AM therapy was started (D2-W0, Fig. 3); 14 AM applications during 30 weeks led to complete wound healing, Fig. 4. All wounds remained healed for more than three years after the end of therapy.

### Case report 3

**Fig. 5:**
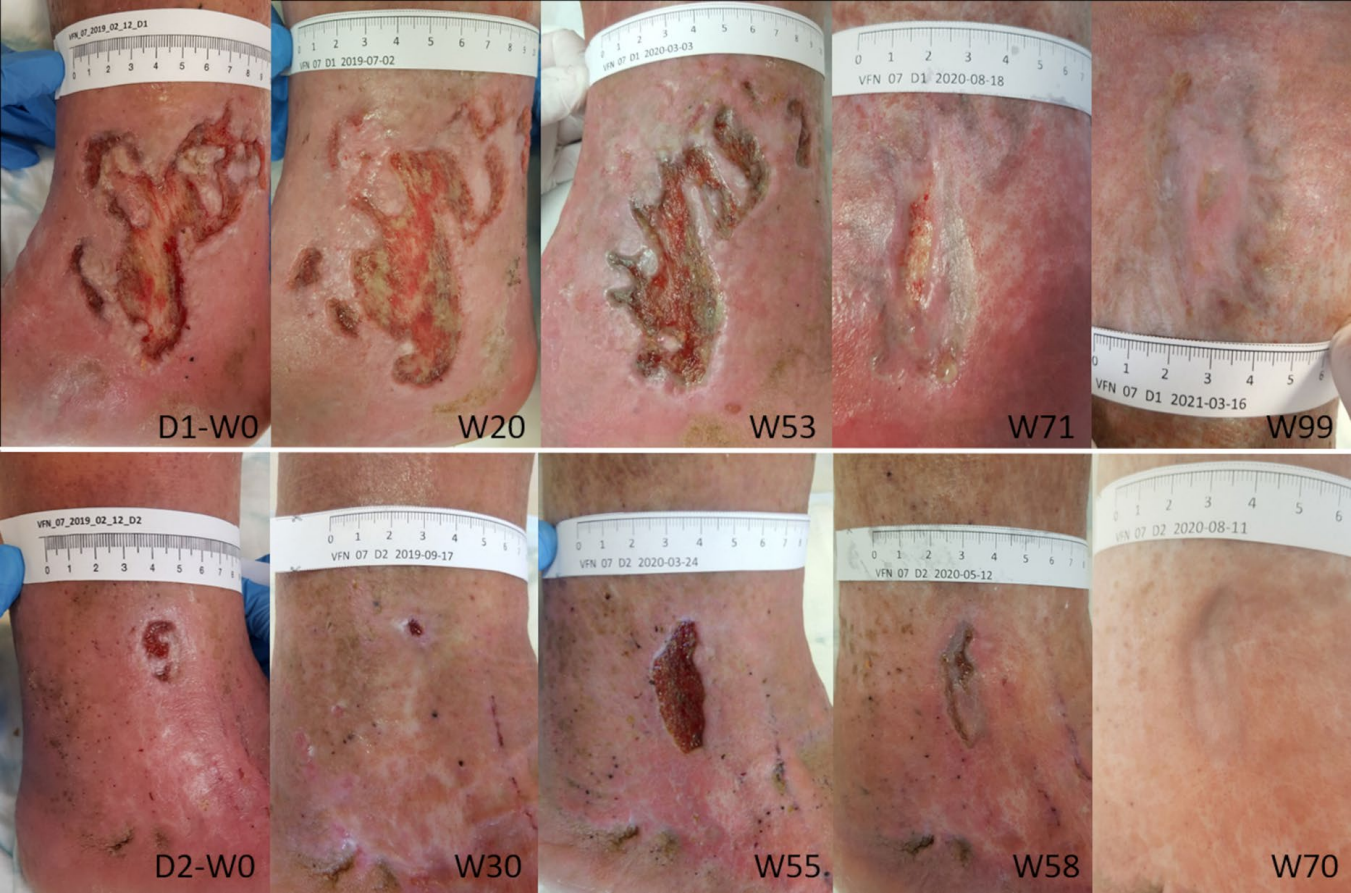
CASE 3, defect D1 and D2. The very slow progress in the healing of D1. W indicates the number of weeks from the beginning of treatment. D2 shows a temporary enlargement (W55) of the treated wound due to infection

A 72-year-old man with two defects on the right lower leg, D1 above the inner ankle and D2 above the outer ankle. Both defects lasted 67 months before the inclusion into AM treatment. At the beginning of treatment, the patient was treated in a different center and was not examined for vascular disease. The patient had a history of arterial hypertension, hyperlipidemia, and chronic atrial fibrillation requiring anticoagulation. The patient’s BMI was 29.3. Vascular examination indicated the closure of Cockett II and III perforators due to their insufficiency. A well-collateralized closure of the heavily calcified superficial femoral artery was also diagnosed, with no indication for intervention. After the perforators were treated, there was only a minimal reduction in the size of the defects despite regular outpatient dressings (Aquacel®Ag). At the time of inclusion in the AM study group, the D1 and D2 areas were 26.8 cm^2^ and 1.27 cm^2^, respectively (Fig. 5), and both were colonized by *P. mirabilis*, respectively *E. coli*, and *S. aureus*. Due to the patient’s PAD history, ankle pressures were measured as part of the initial examination. The examination results and also entering the standard panel of laboratory tests were satisfactory.

**Fig. 6:**
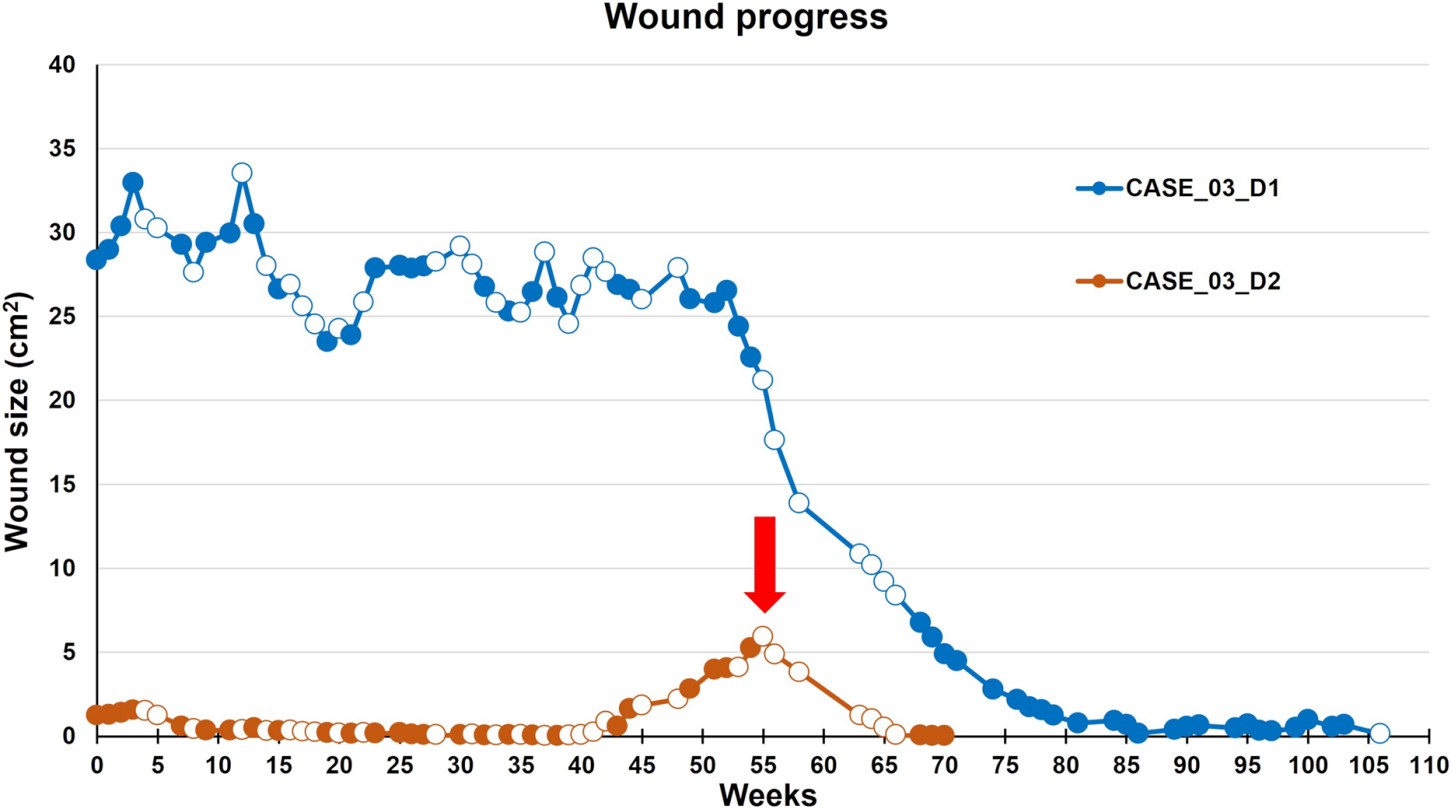
CASE 3. The healing progress of the wound size closure of the patient 3. The temporary enlargement (compared to the base size at W0) of the treated wound is marked by an arrow. Closed and open markers reflect visits with or without amniotic membrane application, respectively

The wounds were dressed according to the protocol, with regular monitoring of the microbiological colonization of the defects. D1 was completely healed after 95 weeks (62 applications of AM), and D2 was completely healed after 67 weeks (34 applications of AM), Fig. 5. The patient’s perception of pain decreased from an initial grade of 5 to 3 and eventually 0 in the first and tenth week of therapy, respectively. The pain completely disappeared from the 10th week of AM application. During the follow-up period between scheduled dressings, the patient’s limb reddened, and body temperature increased with a culture finding of *Streptococcus agalactiae*. The finding required systemic antibiotic therapy and hospitalization of the patient in the dermatology department. The bacterial infection caused a transient stagnation of the defects, Fig. 6. Despite an initially satisfying result of the treatment, the patient had repeated recurrences of the defects in the course of 6–12 months after the end of the AM application due to the progression of PAD. The patient underwent the placement of a femorocrural bypass by allograft and is currently undergoing regular dressings at the university hospital, where defects are still in progress, despite maximum care.

### Case report 4

A 71-year-old obese man (BMI 36.8) with three defects on his left lower limb (D1, D2, D3) was initially treated in another center. He had a history of recanalization of a short segment of the left superficial femoral artery. The patient was not further monitored or screened for vascular disease. His medical history included PAD, arterial hypertension, chronic atrial fibrillation requiring anticoagulation, and chronic obstructive pulmonary disease. The vascular examination showed good patency of the superficial femoral artery with drainage into the three calf arteries. Due to their incompetence, the patient was indicated for closure of the Cockett I, II, and III perforators. After treatment of the perforators, there was only minimal reduction in the defect size despite regular outpatient dressings (Aquacel®Ag). At the time of inclusion in the study for the application of AM, wounds had persisted for 62 months, and the D1, D2, and D3 areas were 25.4 cm^2^, 2.1 cm^2^, and 3.4 cm^2^, respectively. The bacterial colonization included strains of *P. mirabilis, S. aureus*, and *Pseudomonas aeruginosa*. Due to the history of PAD, ankle pressures were measured during the initial evaluation. The examination and also entering the standard panel of laboratory tests were satisfactory.

**Fig. 7:**
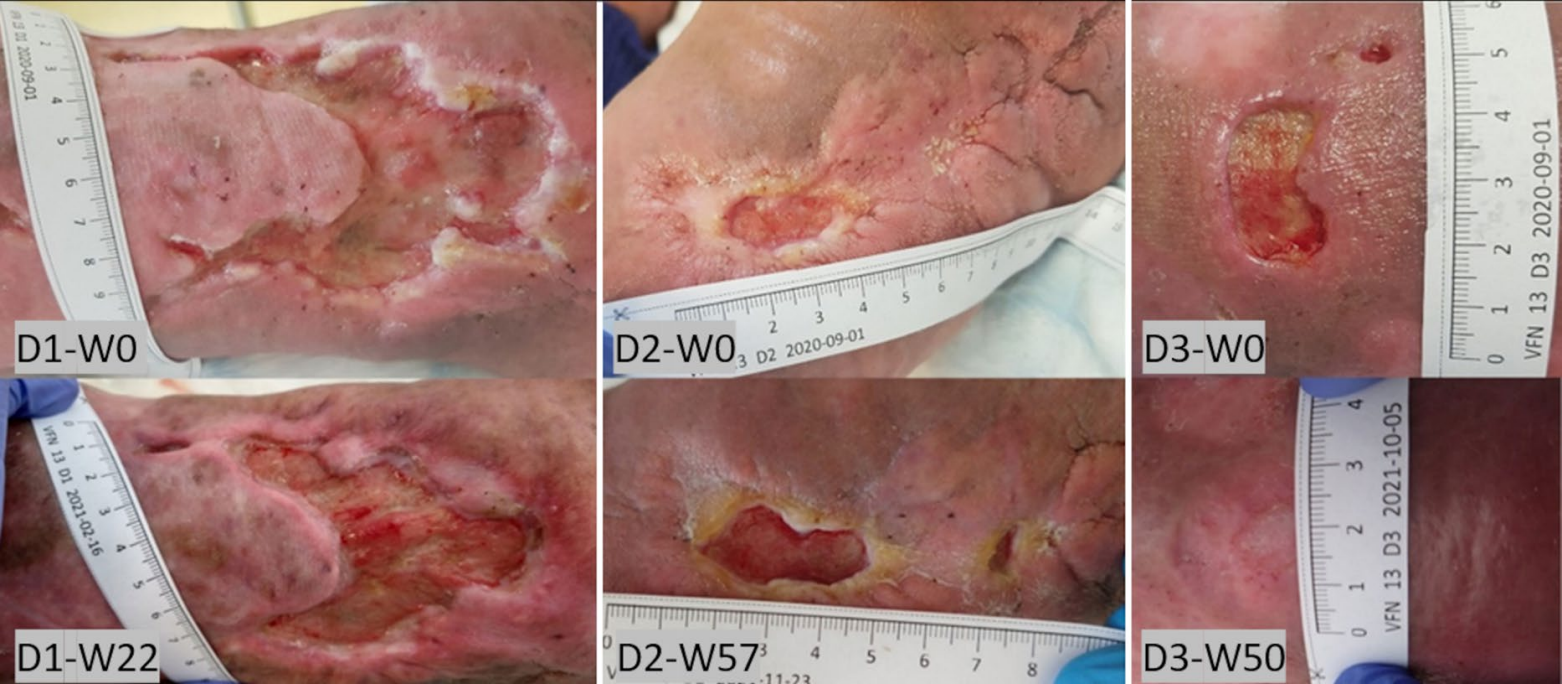
CASE 4. Defects D1, D2, and D3. Out of these three defects, only D3 was healed

**Fig. 8:**
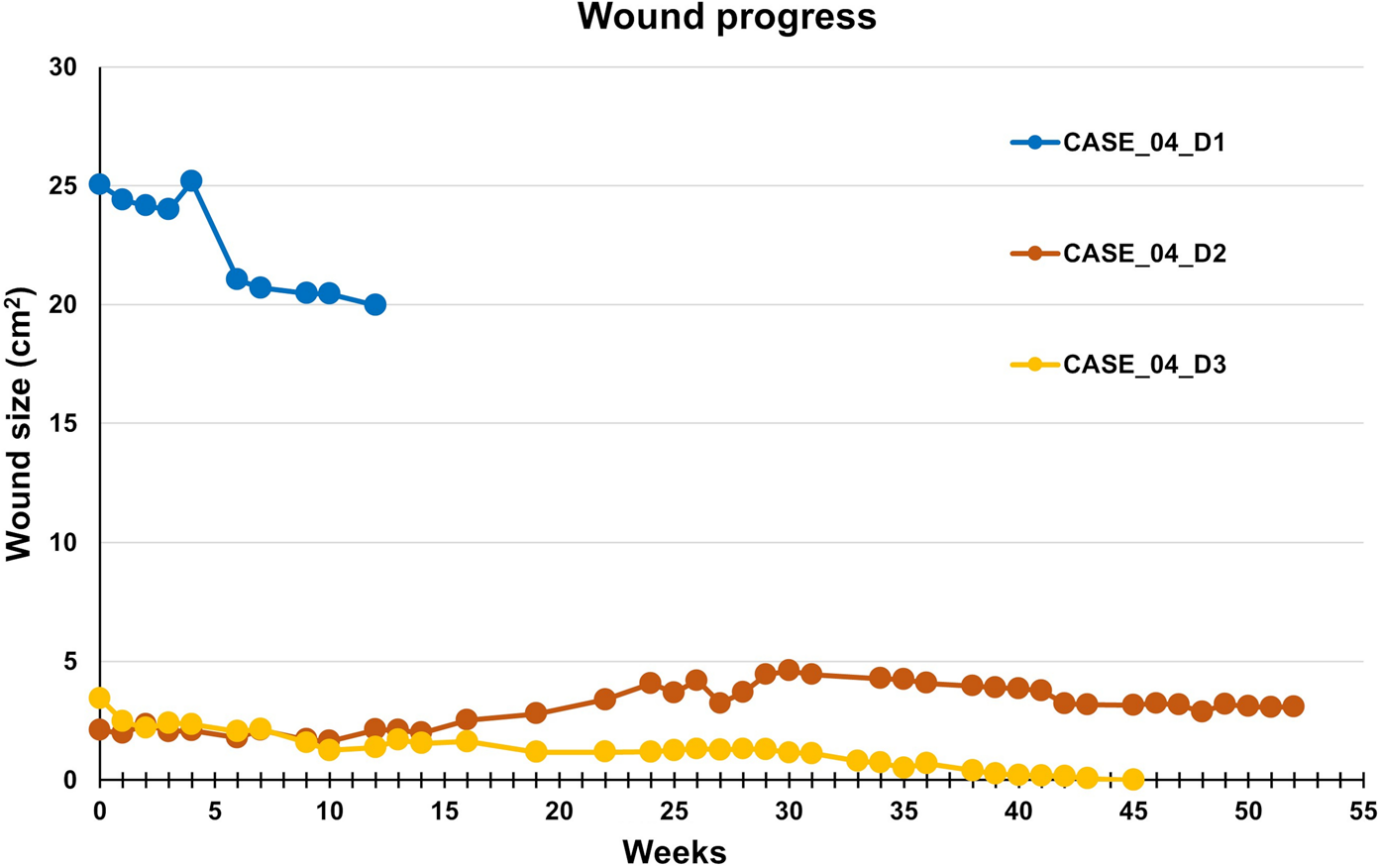
CASE 4. The healing progress of the wound size closure of the patient 3. Closed markers reflect visits with amniotic membrane application

The wounds were dressed according to the protocol, with regular monitoring of the microbiological colonization. During the observation period (12 weeks without AM, then 10 AM applications), the D1 area decreased by 20.3%, while D2 increased by 47.9% after 53 weeks and 45 AM applications. D3 completely healed after 45 weeks (40 AM applications), Fig. 7. The patient experienced a decrease in the perceived pain from the initial grade 2 to grade 1 after the 1st week of AM application, with no further pain relief. Repeated episodes of bacterial infection of the defects during dressings required antibiotic treatment. The patient showed signs of general hygiene neglect and poor wound care. Despite regular home care and psychological intervention, it was not possible to maintain an adequate level of hygiene. The patient often arrived at the clinic with no dressing on the wound, which was often contaminated, so his participation in the project was reduced despite relatively promising results during the first ten weeks, Fig. 8.

For this reason, only two smaller defects (D2, D3) continued to be treated. This led to the complete healing of D3; however, D2 did not respond to the treatment and did not heal. During the 2 years follow-up period, D1 and D2 worsened, while D3 remained healed.

### Case report 5

A 45-year-old woman with a BMI of 28.1, without any internal disease, with an ulcer above the inner ankle of the left lower limb, which developed in combination due to CVI and lymphedema. The defect had been treated in another hospital for an extended period, and the patient had not been evaluated for vascular problems. After examining the arteries and veins of the lower limbs, endovascular treatment of the left saphenous vein and closure of the Cockett II perforator were performed. Despite the treatment of CVI, significant lymphedema persisted even after the procedure. The patient had a history of increased sensitivity to the local application of silver-containing materials and reported strong pain in the defect. After the procedure, a combination of Nu-Gel™ and Xeroform with appropriate compression therapy was used for dressing within the center, but without significant effect. At the time of enrollment into the AM study group, the ulcer was 2.3 cm^2^ in size, and it was colonized by *Enterobacter cloacae*, the surrounding area showed signs of inflammation. However, the level of CRP was normal (4.6 mg/l). Ankle pressure could only be measured at the *a. dorsalis pedis*, inflammation markers were low, and the nutritional status was satisfactory.

**Fig. 9:**
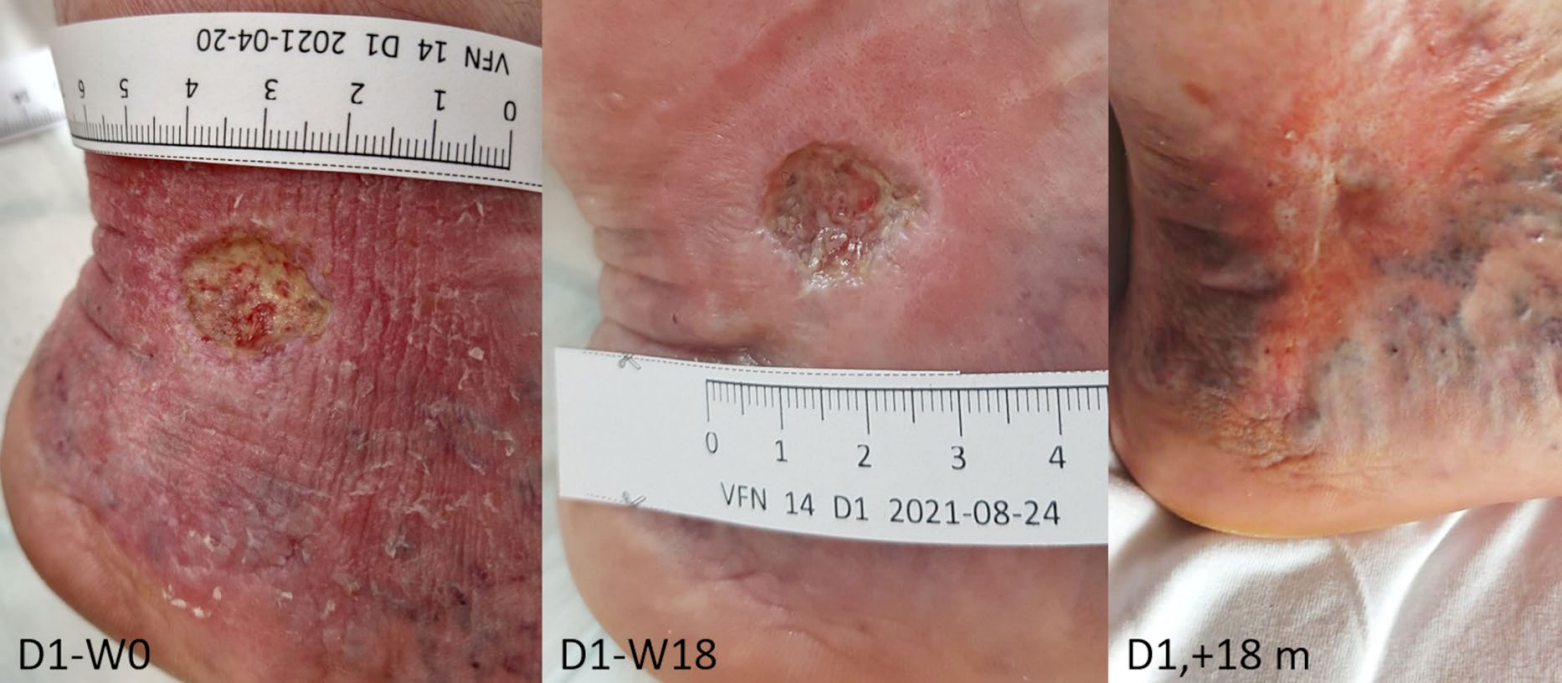
CASE 5. Defect D1 was treated for 20 weeks with no significant wound closure; after repeating endovascular closure of the calf perforators, the wound was closed 18 months (D1, + 18 m) after the end of AM application

**Fig. 10:**
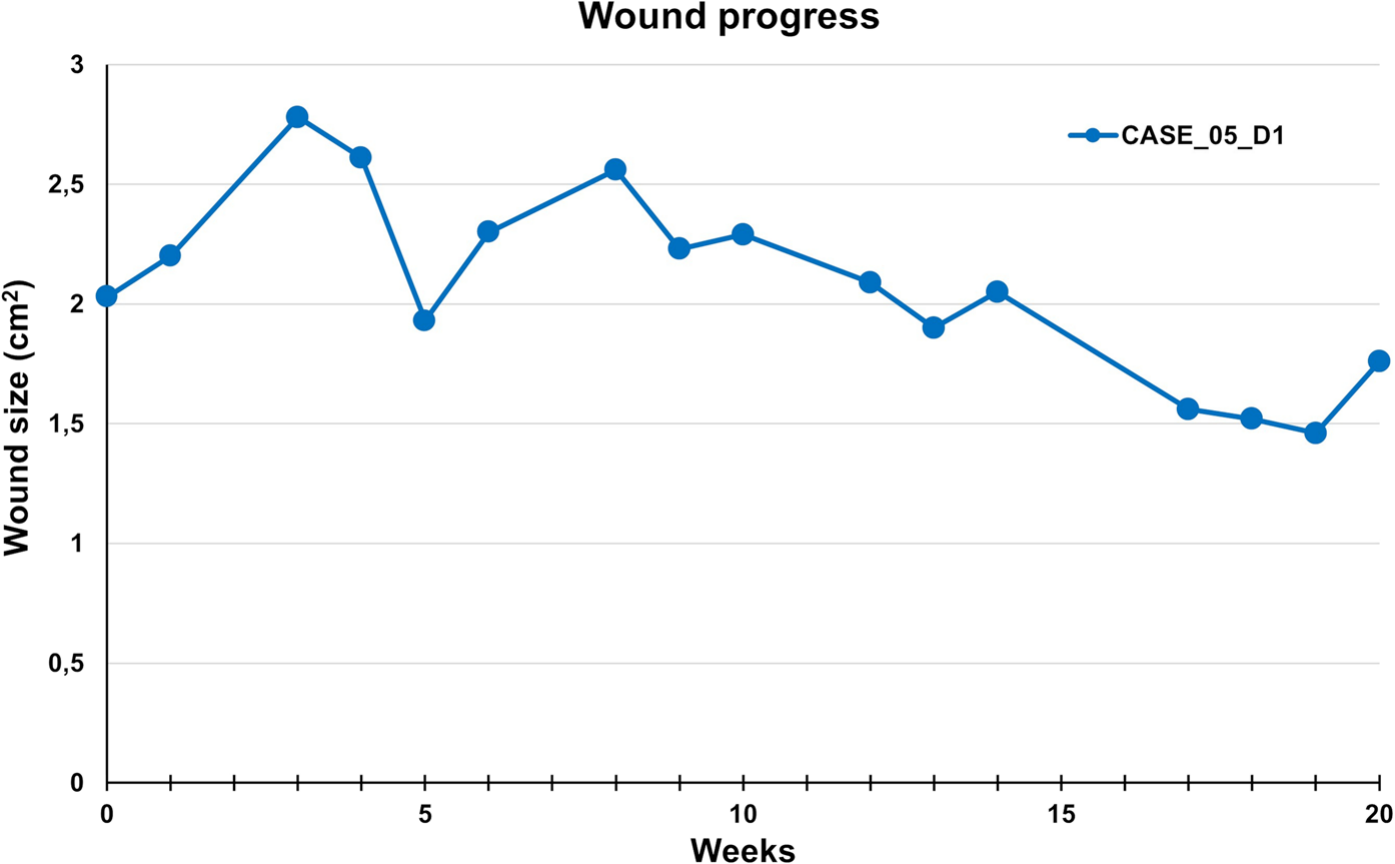
CASE 5. Defect D1 was treated for 20 weeks with no significant wound closure. Wound size oscillated between 1.5 and 3 cm^2^. Closed markers reflect visits with amniotic membrane application

The patient was cared for in accordance with the established protocol, with regular monitoring of the wound microbiological colonization. Despite clear improvement in skin coverage in the surrounding area, the wound showed minimal signs of healing. The patient participated in a 20-week treatment during which she underwent 18 AM applications. The wound size decreased by 13.3% during the monitoring period, Figs. 9 and 10. After the fifth week of AM application, the patient reported significant relief from pain (decrease from grade 5 to 3); from the tenth week of therapy, perceived pain decreased to grade 1 and remained unchanged until the end of the therapy. The AM applications were administered without any local adverse effects. The treatment failure was unexpected in a relatively young patient. It may have been related to torpid edema, particularly in the absence of the possibility of excluding prolonged standing due to her occupation as a salesperson. After six months, the wound remained unhealed, with fluctuating size and a return of pain during dressing changes.

Finally, after repeating endovascular closure of the calf perforators, the wound was closed 17 months after the end of AM application.

## Discussion

Chronic nonhealing wounds are a challenging healthcare issue for healthcare professionals and patients. Providing effective care for these patients is demanding, requiring healthcare professionals to have endurance during frequent dressing changes (Frykberg and Banks 2015). On the other hand, patients must endure reduced quality of life, including pain, discomfort, limited mobility, and the ability to work. These factors can contribute to treatment adherence problems, leading to high costs for the healthcare system (Olsson et al. 2019; Raffetto et al. 2021).

Despite the highly diverse etiology of chronic nonhealing wounds, a consistent approach to their healing can typically be taken once the underlying cause has been identified and effectively addressed. This approach includes a series of treatments to promote wound closure, reduce the risk of infection, prevent a recurrence, facilitate the natural healing process, and promote healthy tissue growth (Eriksson et al. 2022; Frykberg and Banks 2015). Amniotic membrane application has been found to be beneficial in the treatment of such wounds due to its anti-inflammatory, anti-scarring, pain-relieving, and wound-healing properties (Dadkhah Tehrani et al. 2020), and for the treatment particularly cryopreserved (Lavery et al. 2014; Gibbons 2015) and lyophilized allografts are used (Zelen et al. 2015).

To demonstrate the potential effectiveness of AM treatment in promoting the healing of chronic nonhealing wounds, we present a group of five cases, four with primary etiology of CVI and one with PAD (case 4). The purpose of the AM applications was to accelerate the healing process in a site that had already been exhausted by chronic nonhealing in patients expected to have good healing capacity and cooperation. Therefore, a longer healing time and a significant number of AM applications were primarily anticipated. During the regular applications, we noticed two distinct patient groups. The first group comprised three patients (cases 1–3, 6 wounds in total) with multiple comorbidities, who cooperated well and responded well to the AM application. We also observed a very good response in patients with defects purely based on CVI. The second group included two patients (cases 4 and 5, 3 wounds in total) who were expected to heal well despite their medical histories. However, they had poor outcomes due to a history of bacterial colonization, non-compliance with regime restrictions, and inadequate wound care.

In addition to wound healing, our results show that AM treatment has unambiguous analgesic properties, which contribute to improving patient comfort and quality of life (Pesteil et al. 2007; Odet et al. 2022; Svobodova et al. 2023b). In cases 1–3, who were healed completely, the pain decreased markedly within the first several weeks of treatment by AM allografts; the persisting pain relief was also observed in two patients whose wounds were resistant to AM treatment. Patients must adhere to the treatment regimen, care for the wound, and prevent bacterial superinfection to ensure the best possible outcomes.

By exploring these cases in detail, we aim to provide clinicians with a better understanding of how amniotic membrane treatment can promote healing in chronic nonhealing wounds and encourage individualized patient care to treat complex medical conditions. Furthermore, our findings highlight the importance of individualized patient care, as the success of the treatment appears to be closely tied to patient individual factors.

## Conclusion

The highly effective treatment of chronic nonhealing wounds using AM allografts application is progressively becoming standard procedure. Although this is a promising treatment, its effectiveness can be affected by various patient factors. Therefore, it is crucial to apply an individualized treatment plan that takes these factors into account. By tailoring the treatment plan to each patient, treatment effectiveness can be optimized and ultimately improve patient outcomes.
